# Sustainable health: the need for new developmental models

**DOI:** 10.2471/BLT.14.145219

**Published:** 2014-10-01

**Authors:** Iris Borowy

**Affiliations:** a Institute for the History, Theory and Ethics of Medicine, RWTH Aachen University, Wendlingweg 2, 52074 Aachen, Germany.

In 2015, the eight Millennium Development Goals (MDGs) will probably be replaced by 17 Sustainable Development Goals (SDGs). Although only one SDG names health directly, it has been assumed that health involves a broad range of social determinants covered by the other SDGs and that sustainable health requires a sustainable world.

Although any form of development that allows good-quality population health to be sustained far into the future would be very welcome, the SDGs have some inherent contradictions that illustrate the problems faced by those attempting such development. For instance, part of SDG 8 – target 8.1 – calls for “per capita economic growth … and in particular at least 7% per annum GDP [gross domestic product] growth in the least-developed countries” and target 10.1 similarly demands above-average income growth for the poorer 40% of the population.^1^ Given the link between income inequality, poverty and poor health,^2^ it is clear that population health in many places would be improved if these two targets could be met. However, target 9.2, which aims to “promote inclusive and sustainable industrialization, and by 2030 raise significantly industry’s share of employment and GDP … and double its share in LDCs [least developed countries]” elicits concern about the substantial health burden caused by past and present industrialization. It has recently been estimated that emissions – particularly those from industries, transportation and power generation – were responsible for 3.7 million premature deaths in 2012.^3^ How can industry grow – and benefit health by reducing poverty and, hopefully, economic inequality – without increasing the health burden of industrialization?

This challenge has been recognized for some time. In 1997, the World Health Organization (WHO) distinguished between traditional environmental health risks – e.g. indoor air pollution and lack of sanitation – and so-called modern risks – e.g. industrial pollution and climate change.^4^ Meanwhile, recent data confirm that these modern risks appear to be posing a growing threat to health.^5^ In 2005, the Millennium Ecosystem Assessment concluded that climate change, land degradation, biodiversity loss and the depletion and contamination of freshwater had had – or would have – substantial adverse effects on human health. These effects are direct (e.g. increasing floods and heat waves) or indirect, including those mediated through ecosystems (e.g. increased risks of infectious diseases, reduced food yields, loss of livelihood, population displacement and armed conflict). Paradoxically, such adverse effects frequently emanate from changes to ecosystems that were made by humans to meet the growing needs for food and income and that resulted in improved human health in the past.^6^ The post-2015 development model has the challenge of protecting and improving health without simultaneously causing an environmental impact detrimental to human health.

Although the SDGs are much bolder than the MDGs, they may still prove to be insufficient. For example, target 8.4 calls for improvements in global resource efficiency, making use of the 10-year Framework of Programmes on sustainable consumption and production. Bringing consumption and production onto a sustainable level would clearly have health benefits, but it is highly improbable that increased efficiency alone will ever come close to achieving this goal.^7^ In 2000, a far more radical approach was outlined in the charter of the People’s Health Movement, which demanded a transformation of world economic structures and a 90% reduction in consumption and pollution in industrialized countries.^8^ In our current economic system, however, such a reduction would provoke widespread unemployment and impoverishment. The challenge is to find and build consensus on a socioeconomic–environmental development model that would allow the maximization of health benefits while minimizing health costs.

Several authors and agencies have already suggested concepts for transforming global socioeconomic structures into sustainable systems.^9^ Most of these concepts share some common features, such as complementing or replacing GDPs with more informative indicators, changing taxation, trade and banking regulations, aiming at increased resource rather than labour productivity, and translating increased productivity into more free time instead of more material consumption. It is not unknown for health organizations to engage in critical discussions on economic issues. In 2011, for example, WHO made clear that cash transfer programmes affected health and that, therefore, health agencies should participate in the design and development of such programmes.^10^ WHO – and other stake-holders in international health – should now engage in discussions on socioeconomic systems that offer the prospect of sustainable health.
